# Supplementary material for: Chromatin accessibility landscape and active transcription factors in primary human invasive lobular and ductal breast carcinomas
Source: Breast Cancer Res. 2022 Jul 29;24:54. doi: 10.1186/s13058-022-01550-y (PMC9338552; doi:10.1186/s13058-022-01550-y)
Supplement: Supplementary file 1 — Additional file 1. Supplementary Figures and Supplementary Table 3. [file 13058_2022_1550_MOESM1_ESM.docx]

**Chromatin accessibility landscape and active transcription factors in primary human invasive lobular and ductal breast carcinomas**

**Supplementary Figures**


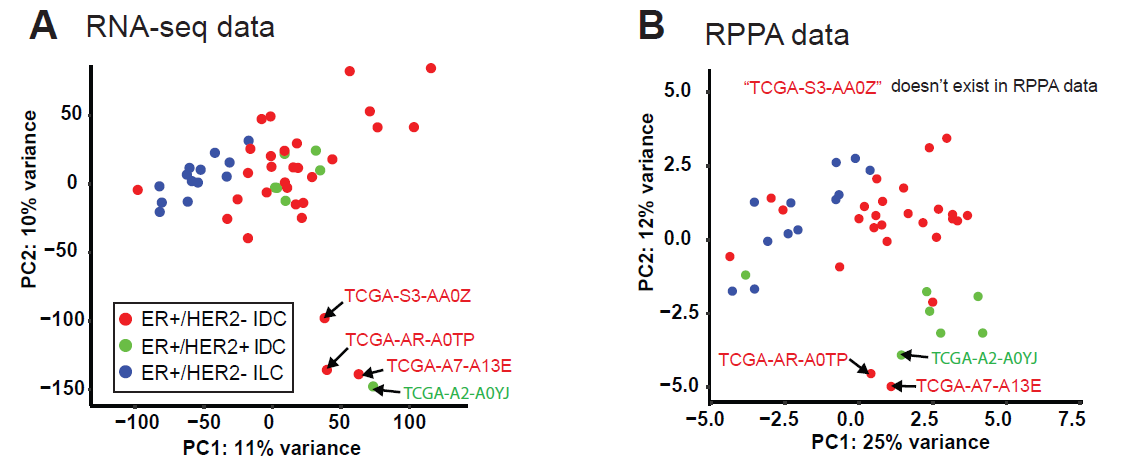


**Supplementary Figure 1.** **PCA of gene and protein expression data. (A)** ER+ tumor clustering by TCGA RNA-seq data (16,418 genes after removing low read count genes) or **(B)** by Reverse Phase Protein Array (RPPA) data (132 proteins).

*
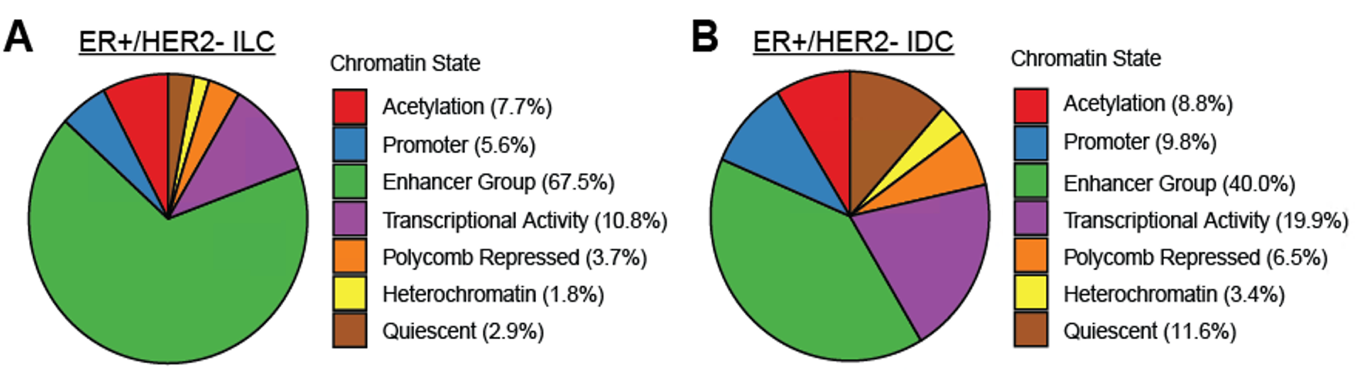
*

**Supplementary Figure 2.** **(A-B)** Pie charts show the percentage of DA ATAC-seq peaks (FDR < 0.05) ILCs vs. IDCs according to the ChromHMM reference of genomic states.

**Supplementary Figure 3.** **Enrichment of TF-binding motifs in promoter or distal intergenic regions for ILCs and IDCs.** **(A-C)** Enrichment of TF-binding motifs per the promoter region (503 peaks), distal intergenic region (2,480 peaks), or intronic region (1,856 peaks) enriched in ILCs. **(C-D)** Enrichment of TF-binding motifs per the promoter region (1,104 peaks), distal intergenic region (2,729 peaks), or intronic region (2,445 peaks) enriched in IDCs. The top 10 most enriched motifs were selected and displayed.


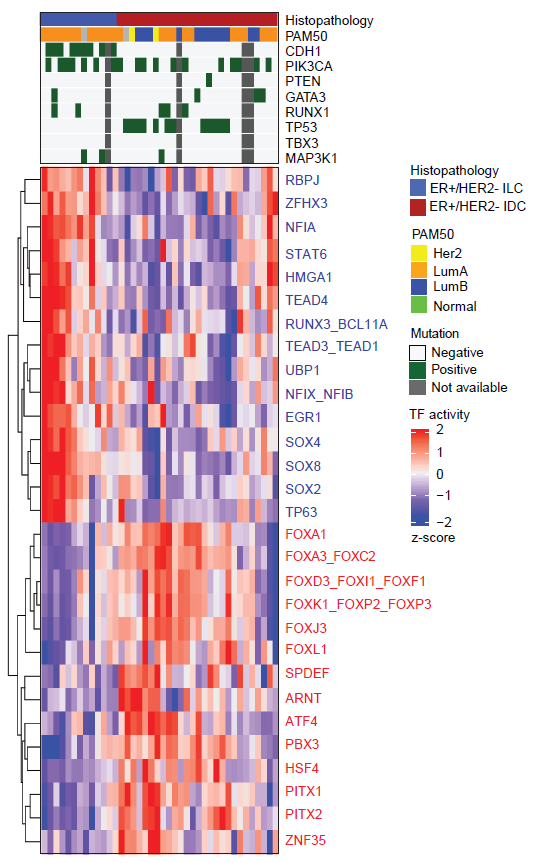


**Supplementary Figure 4.** **Heatmap for TF activities.** TFs significantly associated with ILCs (15 TFs in blue) and IDCs (14 TFs in red) (an absolute mean TF activity difference > 0.035 and the FDR-corrected p-value = 0.05).


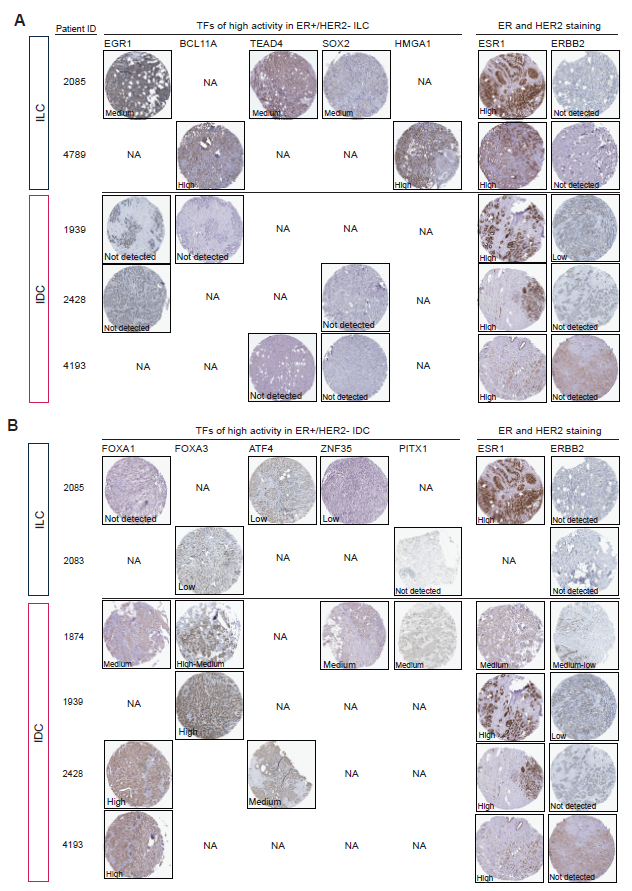


**Supplementary Figure 5.** **Immunohistochemical staining for TF protein expression and corresponding ER and HER2 staining.** **(A)** EGR1, BCL11A, TEAD4, SOX2, and HMGA1, of which high activities in ILCs, have the protein expression high or medium in ILCs, but not detected in IDCs. **(B)** FOXA1, FOX3, ATF4, ZNF35, PITX1, of which high activities in IDCs, have the protein expression high or medium in IDCs, but not detected or low in ILCs. NA, tumor samples of staining images are not available.

**Supplementary Table**

**Supplementary Table 3. List of ER+ ILC or ER+ IDC specific TF genes and essentiality scores derived from siMEM analysis.**

The p-value/FDR derived for each TF according to published screens (GSE73526) using siMEM.

|  | Gene | siMEM score difference | | p-value | FDR |
| --- | --- | --- | --- | --- | --- |
| TFs of high activity in ILCs | **RUNX3** | **-0.694** | | < 1x10^-16^ | 0.002 |
|  | **SOX4** | **-0.264** | | 0.002 | 0.013 |
|  | **TEAD3** | **-0.244** | | 0.015 | 0.054 |
|  | **UBP1** | **-0.392** | | 0.015 | 0.054 |
|  | **NFIA** | **-0.237** | | 0.055 | 0.151 |
|  | **BCL11A** | **-0.173** | | 0.065 | 0.151 |
|  | **TEAD1** | 0.239 | | 0.094 | 0.189 |
|  | STAT6 | -0.138 | | 0.141 | 0.246 |
|  | ZFHX3 | 0.196 | | 0.209 | 0.348 |
|  | RBPJ | 0.195 | | 0.286 | 0.445 |
|  | NFIB | -0.070 | | 0.343 | 0.480 |
|  | NFIX | 0.195 | | 0.388 | 0.494 |
|  | HMGA1 | -0.081 | | 0.550 | 0.642 |
|  | EGR1 | 0.038 | | 0.803 | 0.865 |
|  | TEAD4 | -0.024 | | 0.899 | 0.899 |
| TFs of high activity in IDCs | **FOXA1** | **-0.567** | | < 1x10^-16^ | < 1x10^-16^ |
|  | **SPDEF** | **-0.480** | | 0.001 | 0.008 |
|  | **PBX3** | 0.255 | | 0.010 | 0.052 |
|  | **HSF4** | 0.183 | | 0.051 | 0.192 |
|  | FOXJ3 | 0.099 | | 0.106 | 0.317 |
|  | FOXP3 | 0.098 | | 0.137 | 0.343 |
|  | FOXF1 | 0.127 | | 0.181 | 0.388 |
|  | FOXC2 | -0.095 | | 0.263 | 0.494 |
|  | PITX1 | -0.048 | | 0.323 | 0.538 |
|  | PITX2 | 0.037 | | 0.538 | 0.808 |
|  | ATF4 | 0.004 | | 0.968 | 0.993 |
|  | FOXI1 | -0.018 | | 0.815 | 0.993 |
|  | FOXL1 | 0.004 | | 0.962 | 0.993 |
|  | ZNF35 | 0.000 | | 0.993 | 0.993 |
|  | FOXD3 | 0.027 | | 0.778 | 0.993 |
| o Yellow highlight: FDR < 0.2 | | | |  |  |
| o Blue text: Essential genes in ER+ ILC cell lines | | | |  |  |
| o Red text: Essential genes in ER+ IDC cell lines | | | |  |  |
